# Changes in Exosome Release in Thyroid Cancer Cells after Prolonged Exposure to Real Microgravity in Space

**DOI:** 10.3390/ijms22042132

**Published:** 2021-02-21

**Authors:** Petra M. Wise, Paolo Neviani, Stefan Riwaldt, Thomas Juhl Corydon, Markus Wehland, Markus Braun, Marcus Krüger, Manfred Infanger, Daniela Grimm

**Affiliations:** 1 The Saban Research Institute, Children’s Hospital Los Angeles, University of Southern California, 4650 Sunset Blvd, Los Angeles, CA 90027, USA; wisepetra@gmail.com (P.M.W.); pneviani@chla.usc.edu (P.N.); 2 Department of Microgravity and Translational Regenerative Medicine, Clinic for Plastic, Aesthetic and Hand Surgery, Otto von Guericke University, Universitätsplatz 2, 39106 Magdeburg, Germany; stefan.riwaldt@med.ovgu.de (S.R.); markus.wehland@med.ovgu.de (M.W.); marcus.krueger@med.ovgu.de (M.K.); manfred.infanger@med.ovgu.de (M.I.); 3 Department of Biomedicine, Aarhus University, Ole Worms Allé 4, 8000 Aarhus C, Denmark; corydon@biomed.au.dk; 4 Department of Ophthalmology, Aarhus University Hospital, Palle Juul-Jensens Blvd. 99, 8200 Aarhus N, Denmark; 5 Research Group "Magdeburger Arbeitsgemeinschaft für Forschung unter Raumfahrt- und Schwerelosigkeitsbedingungen" (MARS), Otto von Guericke University, 39106 Magdeburg, Germany; 6 Deutsches Zentrum für Luft- und Raumfahrt (DLR), Raumfahrtmanagement Bonn-Oberkassel, 53227 Bonn, Germany; m.braun@dlr.de

**Keywords:** thyroid cancer, cell culture, exosomes, microgravity, spaceflight, transmembrane proteins, tetraspanins

## Abstract

Space travel has always been the man’s ultimate destination. With the ability of spaceflight though, came the realization that exposure to microgravity has lasting effects on the human body. To counteract these, many studies were and are undertaken, on multiple levels. Changes in cell growth, gene, and protein expression have been described in different models on Earth and in space. Extracellular vesicles, and in particular exosomes, are important cell-cell communicators, being secreted from almost all the cells and therefore, are a perfect target to further investigate the underlying reasons of the organism’s adaptations to microgravity. Here, we studied supernatants harvested from the CellBox-1 experiment, which featured human thyroid cancer cells flown to the International Space Station during the SpaceX CRS-3 cargo mission. The initial results show differences in the number of secreted exosomes, as well as in the distribution of subpopulations in regards to their surface protein expression. Notably, alteration of their population regarding the tetraspanin surface expression was observed. This is a promising step into a new area of microgravity research and will potentially lead to the discovery of new biomarkers and pathways of cellular cross-talk.

## 1. Introduction

Man has always looked to the stars, and throughout history has been on the pursuit to reach them. As the technology evolved, mankind took step after step towards this quest, and today space travel for research missions is a reality, with commercial travel and exploration of other planets on the horizon [1,2,3,4]. In addition to the technical challenges space travelers face, the human body itself suffers negative effects from a prolonged lack of exposure to gravitational forces, which can lead to various impairments in multiple physiological systems [5,6,7,8,9,10]. Since the early days of spaceflight, researchers have investigated the effects of weightlessness on the human body [11,12,13].

As spaceflights are rare and require extensive preparation and funding, most of the initial cell biological experiments are conducted under simulated microgravity conditions. Various devices have been developed to successfully simulate weightlessness on Earth, such as the random positioning machine (RPM) and clinostats [14,15,16,17,18]. The observed changes after cell incubation within these devices often predict cellular changes during spaceflight, but by direct comparison simulated and real microgravity do not cause identical effects [19]. Therefore, our team took the opportunity to conduct experiments under prolonged microgravity as part of a joint enterprise by the German Aerospace Center (Deutsches Zentrum für Luft- und Raumfahrt, DLR) and the Chinese Manned Space Engineering Office (CMSEO) [20] during the Shenzhou-8/SimBox Spaceflight mission in 2011. The planning and execution of cell culture experiments on the unmanned Shenzhou-8 spacecraft was a major challenge. The cell culture equipment had to be created to withstand the physical forces during the mission, as well as being adapted to small, automated modules to enable fluid exchanges [21,22].

Based on hardware tests prior to and during the Shenzhou-8 mission, the results and insights gained through the analysis of the harvested samples provided us with the requisite knowledge to set up another experiment under prolonged exposure to microgravity. We previously demonstrated changes of cancer cells from two- to three-dimensional growth after exposure to simulated microgravity on the RPM [21,22]. Concurrently, genomic adaptations in pathways involved in the cytoskeletal composition were identified [23,24,25]. As described in Section 4.2., the human follicular thyroid cancer cell line FTC-133 was flown to the International Space Station (ISS) on the SpaceX CRS-3 cargo mission in newly designed, automated CellBox-1 modules. Incubation aboard the ISS proceeded for 12 days, following a pre-determined feeding and fixation schedule (flight module, FM). Concurrently, cells from the same passage were grown under the exact same conditions considering the hardware, temperature, and fluid exchange schedule in a laboratory on Earth (ground module, GM). The harvested cells and supernatant were analyzed to determine the growth behavior, adaptations in gene and protein expression, and protein interactions.

To further explore the underlying processes of any differential protein expression, we decided to investigate the cell culture supernatants as to their content of extracellular vesicles (EVs). EVs have evolved into a fast-growing focal point in biomedical research over the last decade, as they show a high potential as biomarkers and in clinical applications due to their emerging roles in disease, as well as normal physiology. EVs are a family of membrane vesicles containing a phospholipid bilayer, which are secreted into the extracellular environment by the majority of (if not all) cells [26,27,28]. These vesicles can be categorized into three major types: Exosomes, microvesicles, and apoptotic bodies. They are found in circulating body fluids, including blood, saliva, and urine [26,29,30,31,32]. EVs reflect their cell of origin with respect to protein, nucleic acid, and lipid content, functionally they mediate cell-cell communication. EVs have the potential to serve as biomarkers for a wide array of diseases in clinical diagnosis, and may lead the way to a variety of future therapeutic approaches. Hence, the role of EVs in the tumor environment has been studied extensively over the last decade. They are transferred from tumors to the surrounding extracellular environment and thus, modulate the immune and therapy response, and promote matrix remodeling as well as angiogenesis [33,34,35,36]. Conversely, the transfer of EVs from the tumor microenvironment to tumor cells enhances tumorigenesis by increasing tumor cell proliferation, resistance to chemotherapy, and migration [36,37,38,39,40,41]. Furthermore, EVs can travel to distant areas of the organism, where they can be involved in the preparation of the pre-metastatic niche [42,43,44,45,46,47].

**Figure 1:**
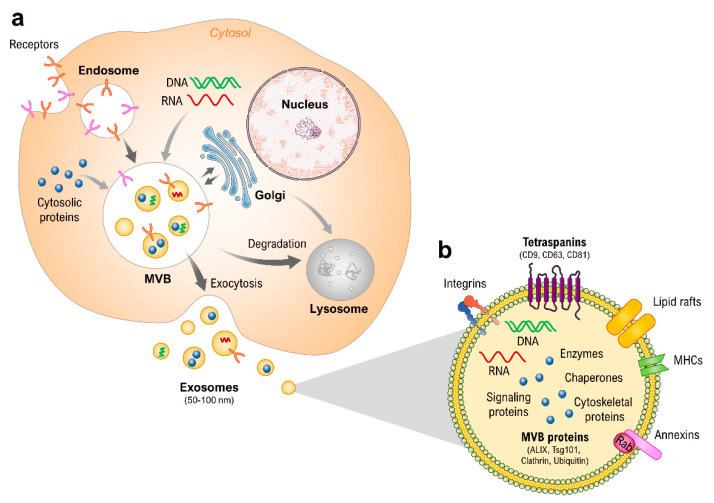
(**a**) Schematic overview of exosome biogenesis, cargo loading, and release. (**b**) Typical molecular contents of an exosome. Parts of the figure are drawn using pictures from Servier Medical Art (https://smart.servier.com (accessed on 20 February 2021)), licensed under a Creative Commons Attribution 3.0 Unported License (https://creativecommons.org/licenses/by/3.0 (accessed on 20 February 2021)).

This research paper will focus particularly on exosomes, which can be distinguished from the remaining EVs by their biogenesis and size. Exosomes range in size from roughly 30 to 150 nm and are further defined by the presence of specific transmembranes, extracellular, and cytosolic proteins, as well as the absence of some intracellular proteins present in other EVs. The International Society for Extracellular Vesicles (ISEV) has detailed the minimal experimental requirements for a definition of exosomes and other EVs [48,49]. Additionally, exosomes are defined by their release mechanism to the extracellular environment via fusion of late endosomes/multivesicular bodies (MVBs) with the plasma membrane (Figure 1) [50,51]. The study and the development of applications of exosomes has so far been challenging, as the initial isolation and enrichment methods resulted in low yields and/or co-precipitation of aggregated and bound proteins, as well as due to their small size. Therefore, finding new ways to characterize these promising vesicles is a focus of researchers worldwide. One of the most common methods of determining the size distribution and concentration of exosomes is nanoparticle tracking analysis (NTA), where the particle size of isolated EVs is calculated by the Brownian motion [52,53]. However, conventional NTA reaches its limit when it comes to determining the cell origin and distinguishing between different EVs. The Western blot analysis is frequently used to verify that isolated vesicles are in fact exosomes, but this requires a rather large number of vesicles. The immunocapture of exosomes on antibody-coated beads followed by the analysis via flow cytometry enhances the specificity of the characterization, but has a slow throughput and is quite costly, particularly with multiplexing for several markers [53,54,55]. Here, we have employed a method based on the single particle interferometric reflectance imaging sensor (SP-IRIS) (ExoView™, NanoView Biosciences, Boston, MA, USA) which allows multiplexed phenotyping and digital counting of various populations of individual exosomes captured on a microarray-based chip. This method allows the characterization of exosomes directly from human biofluids without the need of prior vesicle isolation or concentration, and so allows a detailed analysis with a very small sample size [53,55]. In this paper, we are reporting the results of the characterization of the cell supernatants from samples that were grown under prolonged microgravity on the ISS, as well as their control counterparts incubated on Earth. Given the growing body of knowledge regarding the role of exosomes as biomarkers, as well as their potential in disease diagnostics and therapy, these initial investigations could be the start of a new area of research into understanding adaptations and changes of an organism in space on the level of cellular cross-talk. Additionally, through exosomes we may gain further insights into the mechanisms of cell regulation via EVs.

## 2. Results

Since the first description of secreted membrane vesicles, there has been rapid growth in the number of studies on EVs [56,57]. Over the years, the functional roles of these vesicles have been explored, and with rising interest in the implications and possibilities of EVs many isolation methods have been developed, with the specific method depending on the source material, as well as the requirements needed to explore the given hypothesis [49,54,58,59]. For example, differential centrifugation protocols, as well as methods for both filtration and polymer precipitation have been shown to drastically differ in yield, purity, and functionality of the harvested EVs [58,60,61]. Similarly, the definition and description of EVs has varied greatly and proved overwhelming at times. Therefore, the ISEV suggested that investigators report the amount of several proteins (three or more) in at least a semi-quantitative manner in any EV preparation, including EV isolates obtained from body fluids or from secreting cells in vitro. The proteins described and characterized should be proteins expected to be present in the EVs of interest, especially transmembrane proteins and cytosolic proteins with membrane-binding capacity. In addition, the level of presence of proteins not expected to be enriched in EVs of endosomal origin should also be determined [48]. Transmembrane proteins expected to be present in exosomal preparations include, among others, tetraspanins (CD9, CD63, CD81), integrins, and growth factor receptors. The group of expected or enriched cytosolic proteins includes endosome or membrane-binding proteins (TSG101) and signal transduction or scaffolding proteins (syntenin). Intracellular proteins that are absent or under-represented in exosomes, but present in other types of EVs, are proteins found in the endoplasmic reticulum (ER) (calnexin) or the Golgi apparatus (GM130) [48].

The method of this study was SP-IRIS, using the ExoView™ system (NanoView Biosciences, Boston, MA, USA). With this method, human exosomes can be selectively captured on a chip plate directly from biofluids without prior need to isolate and concentrate EVs. Additionally, the method is suitable for even very small sample sizes, as high as 20 µL for unprocessed not concentrated samples. To assess the amount and population distribution of exosomes in the cell supernatants harvested from the CellBox-1 experiment, we used EV-TETRA-C ExoView Tetraspanin chip plates (NanoView Biosciences, Boston, MA, USA), which capture exosomes via pre-loaded antibodies (ABs) to the tetraspanins CD9, CD63, and CD81. The interferometric analysis enables the researcher to investigate the particle count and the size distribution of a given sample [53,55]. By counter-staining the captured exosomes with fluorescent ABs to the three tetraspanins it is possible to characterize the EVs according to their transmembrane protein expression, and identify different populations present in the sample. Here, we describe the results gained from the GM and FM cell culture supernatants harvested from the CellBox-1 experiment on the ISS.

### 2.1. Interferometric Analysis

#### 2.1.1. Particle Concentration

**Figure 2:**
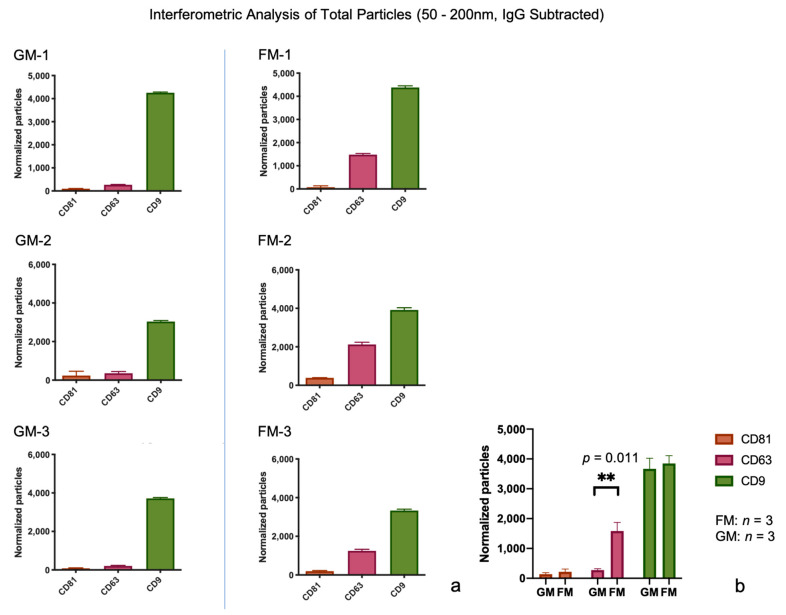
(**a**) Total count of captured particles via the interferometric analysis, from both sample sets, ground module (GM) and flight module (FM). Measurements were taken in triplicates, values are shown with the standard deviation (SD). (**b**) The statistical analysis via the unpaired *t*-test was performed to compare the GM group to the FM group on different card spots. Statistical significance is displayed as ** (*p* ≤ 0.01).

The absolute number of captured particles was measured via interferometric analyses, by scanning the tetraspanin spots (CD9, CD63, and CD81) as well as the IgG control, all in triplicate experiments. As pictured in Figure 2, the majority of EVs were bound to the DC9 spots in both the GM and FM samples (GM: 4256/3038/3715.33 particles; FM: 4380.5/3915/3331 particles), followed by particles bound to CD63 (GM: 268/355/205.66 particles; FM: 1479.5/2121.5/1245 particles), and CD81 (GM: 98.33/239/87.33 particles; FM: 75/386.5/196.33 particles). The number of CD9 particles is fairly similar in all the samples regardless of the exposure to microgravity, but the numbers of CD63 and CD81 is elevated in FM samples roughly 10-fold (CD63) to 20-fold (CD81), respectively, compared to the GM samples. The total concentration in FM samples averages at 7235.2 particles, and in GM samples at 4094.4 particles, which indicates an average increase in EVs of 74% (Figure 2a, Figure S1). The statistical analysis using an unpaired t-test revealed a significant change in between one group, the comparison of captured particles on the CD63 spot with a *p*-value of 0.011 (Figure 2b).

#### 2.1.2. Particle Size Distribution 

**Figure 3:**
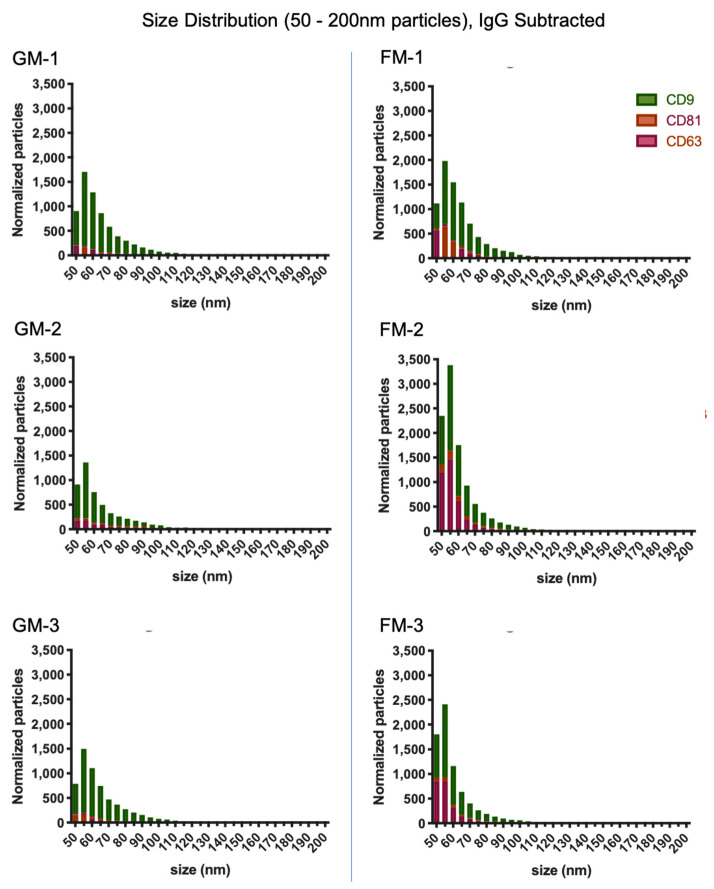
Particle size distribution by the interferometric analysis of both samples sets. Measurements were taken in triplicates; the results were normalized with the IgG control, the size range spans from 50–200 nm.

The size distribution of the captured EVs was determined by interferometry, as well. Currently, the ExoView interferometric analysis is limited to the size range of 50–200 nm. As shown in Figure 3, the majority of particles captured by the tetraspanins are well within the size range of 30–100 nm, which is the defined size of exosomes [48,54] in all GM and FM samples alike. This size range covers an average of 5742 particles in the GM group and 8368 particles in the FM group. Only very few particles were found in sizes larger than 100 nm: On average, 314 particles in GM samples and 252 particles in FM samples. The mode size of exosomes is 60 nm, with an average of 2592 particles in the FM samples and 1520 particles in the GM samples (Table 1 and Table 2). The size distribution on all six analyzed samples shows that the analyzed particles can be considered exosomes, both due to their size and the presence of the tetraspanins CD9, CD63, and CD81 (Figure 3).

### 2.2. Fluorescent Analysis

#### 2.2.1. Total Fluorescent Particle Counts

**Figure 4:**
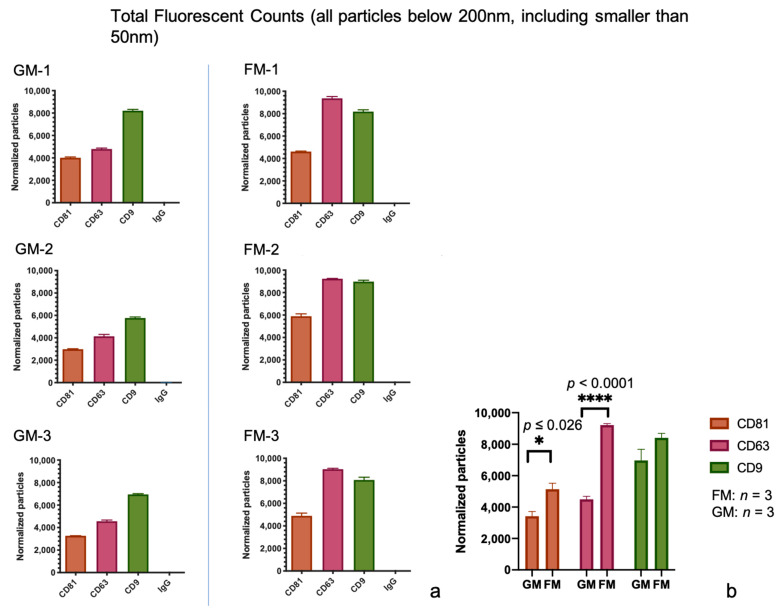
(**a**) Absolute particle counts by fluorimetric scans. Included are all the size particles up to 200 nm, values are shown with SD. (**b**) The statistical analysis via the unpaired t-test was performed to compare the GM group to the FM group on different card spots. Statistical significance is displayed as * (*p* ≤ 0.05) and **** (*p* ≤ 0.0001).

The analysis of the chip plate captured exosomes counterstained with fluorescent antibodies to the tetraspanins offers deeper insights into the varying population of EVs in a given sample, as well as their distribution and changes under different conditions and the ability to detect vesicles below the detection limit of 50 nm. The results of our examination of the total fluorescent particle counts with all three ABs on all capture spots can be seen in Figure 4. The figure displays the combined values of all three fluorescent tetraspanin ABs. It can be observed that in the GM samples, the majority of the particles are found at the CD9 slot (on average 7107/6917/6890 particles), followed by the particles on the CD63 slot (on average 4556/4296/4549 particles), and lastly by CD81 (on average 3470/3368/3416 particles). Contrary to this, the results in the FM samples point to the highest count of bound particles on the CD63 spot (on average 9216/9136/9277 particles), which is a 103% increase compared to the averaged GM samples. The particles on the CD9 spot (on average 8461/8546/8230 particles) display a 21% increase, and the particles on CD81 (on average 5179/5189/5035 particles) a 50% increase in comparison to GM. Looking at the sum of particles, this is a 53% increase over all three spots in the samples that were subjected to prolonged microgravity (Figure 4a). A comparison of all the samples from the GM and FM group via unpaired *t*-tests revealed significant changes in the particles captured on the CD81 spot (*p* ≤ 0.026) and the CD63 spot (*p* < 0.0001) (Figure 4b).

**Figure 5:**
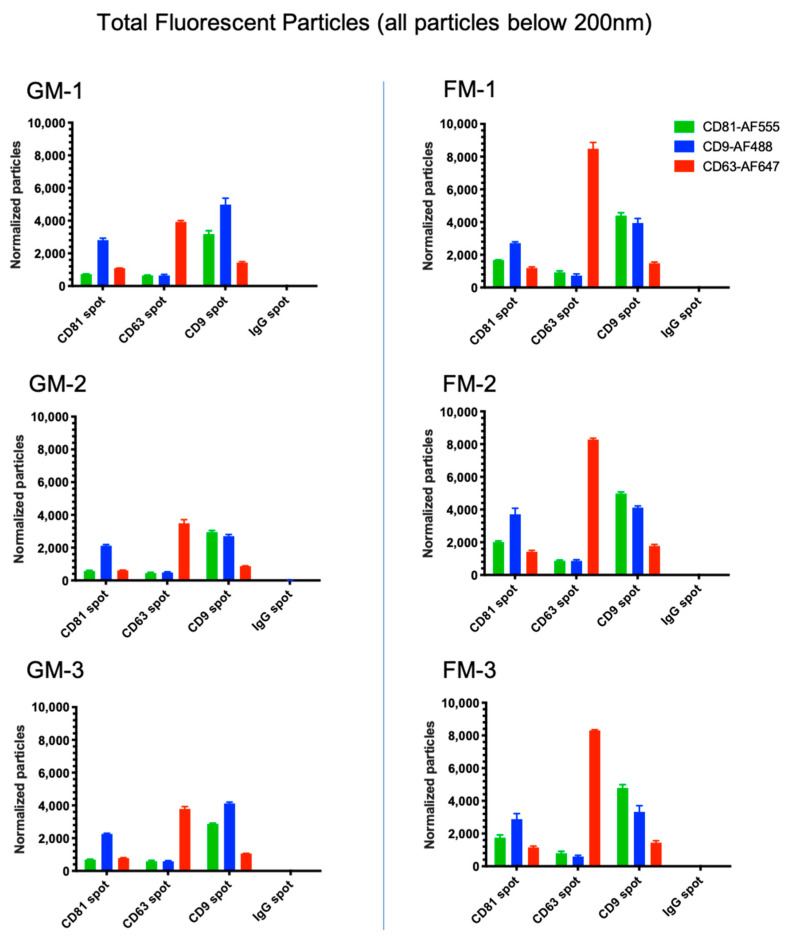
Total particle counts by the fluorimetric analysis up to 200 nm, including particles smaller than 50 nm. Particles were counted for all three counterstains, CD9, CD63, and CD81 separately on the specific spots on the chip plates in triplicates. Values are shown with SD.

One might expect to find the highest particle counts where the AB concurs with the coating of a spot, due to a higher affinity of a particle to a specific slot depending on the surface expression of the protein in question. This only seems to hold true for CD63 in our samples. In both sets, GM and FM, CD63 shows the most prominent count on its own spot on the chip plate and shows lower counts on the remaining spots. CD81, on the other hand, shows the most prominent counts on the CD9 spots in both the FM and GM sample sets. In both sample sets, CD81 shows more particles on the CD9 spot than on the other two, even the CD81 spot itself (Figure 5). A proposed explanation of this discrepancy may be due to the low antigen density on single vesicles (e.g., less than three epitopes available for recognition by the fluorescently conjugated antibody or due to steric hindrance (low epitope accessibility due to the small size of the vesicles).

#### 2.2.2. Colocalization Analysis

##### Single Tetraspanin Surface Expression—CD9, CD63, and CD81

**Figure 6:**
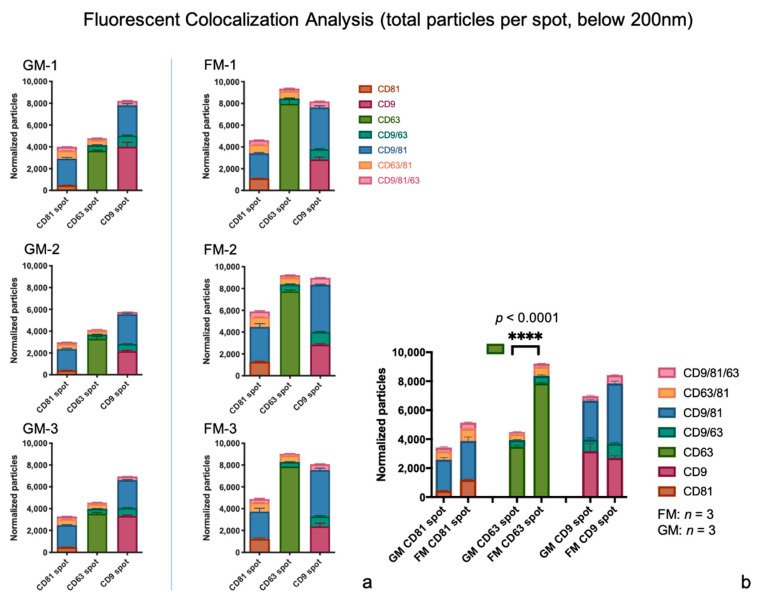
(**a**,**b**) The colocalization analysis of all captured particles. The particles were scanned for single expression of CD9, CD63, and CD81, as well the co-expression of two or all three tetraspanins, values are shown with SD. (**a**) The total particles by spot and per sample, displayed as actual counts. (**b**) The total fluorescent count of particles displayed as a comparison of the two sample groups combined, the displayed median of actual counts. Statistical significance is displayed as **** (*p* ≤ 0.0001).

The number of particles with CD9 surface expression only is slightly elevated in the GM sample set (3169 particles on average) in comparison to the FMs (2696 particles on average, Figure 6a,b; Tables S1 and S2). The population of CD63 only expressing exosomes is by far the largest in both experimental groups, with an average of 3491 particles in the GM sample set and 7856 particles in the FM samples. The statistical analysis of the GM and FM group via unpaired t-tests revealed significant changes in the particles expressing CD63 only (*P* < 0.0001) (Figure 6b). CD81 as a single expressed tetraspanin describes the smallest of the single expression populations with an average of 448 (GMs), respectively and 1198 particles (FMs) on average.

##### Co-expression of two Tetraspanins – CD9/CD63, CD9/CD81, and CD63/CD81

**Figure 7:**
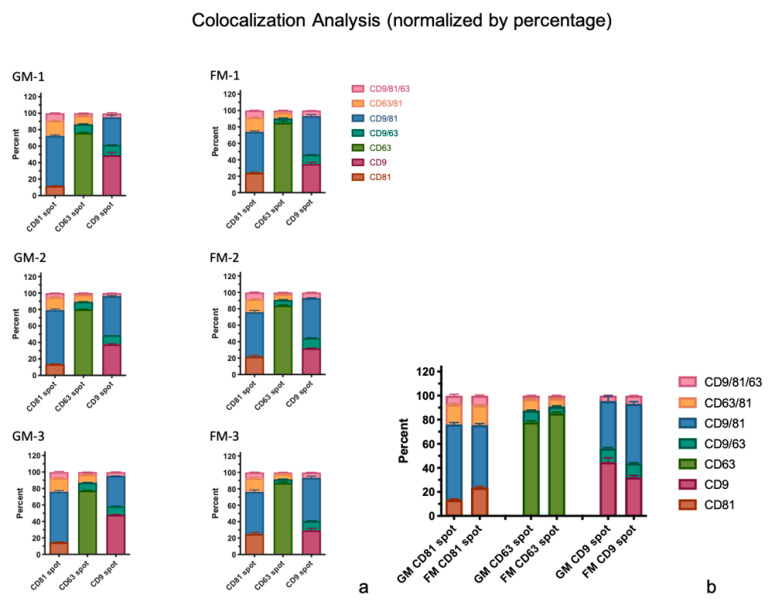
(**a**,**b**) The colocalization analysis of all captured particles. The particle scanned was for a single expression of CD9, CD63, and CD81, as well the co-expression of two or all three tetraspanins, values are shown with SD. (**a**) Total particles by spot and per sample, displayed as a percentage. (**b**) The total fluorescent count of particles displayed in comparison of the two sample groups combined, displayed as a median percentage.

Exosomes co-expressing the tetraspanins CD9 and CD63 only differ slightly in the two experimental sets (GM and FM). In the GMs, 1243 particles were counted, in the FMs 1504 particles. Not unexpectedly, the CD9/CD81 co-expressing exosomes are the largest population of this group, with counts of 4820 particles in the GMs on average, and 6826 particles in the FMs. The CD63/CD81 population is the smallest in this group, averaging 1007 particles in GM and 1476 particles in FM (Figure 6a,b and Figure 7a,b; Tables S1 and S2).

##### Co-Expression of all three Tetraspanins – CD9/CD63/CD81

Exosomes found with all three tetraspanins make up the second smallest population of all, with 704 (GMs) and 1202 (FMs) counted exosomes (Figure 6a,b and Figure 7a,b; Tables S1 and S2).

## 3. Discussion

Since the dawn of manned spaceflight, particular attention has been given to the effects of microgravity on the human organism. These effects are extensive and span from physiological changes and adaptations all the way to altered gene expression and cellular modifications. Physiological adaptations to the cardiovascular, musculoskeletal, and sensorimotor systems and the implications of varying durations of spaceflight thereon have been studied extensively, as well as methods for the preparation and reconditioning of space travelers [62,63,64]. Muscle and bone loss are commonly experienced even with regular exercise prior to and during flights, and these changes can be demonstrated on both the physiological and cellular level [6,8,16].

In recent years, the importance of EVs in intercellular communication and post-transcriptional proteomic regulation, as well as disease biomarkers has been described in detail in various settings [29,65,66,67,68]. The analysis of surface markers has emerged as one defining part of EV and exosome definition and nomenclature [48,49]. With the evolving development of new analytical methods, the possibilities to study in-depth changes in EV secretion, cargo, and population setup have increased significantly. Hence, we took this opportunity to get a view of the results of the CellBox-1 experiments from a different angle. Here, we describe initial experiments to evaluate possible changes in exosomal secretion and populations under prolonged exposure to microgravity in a human follicular thyroid cancer cell line (FTC-133), in which our group has previously studied in various conditions of weightlessness [22,23,24,69,70]. Through our knowledge of the genomic and proteomic changes of this particular cell line, as well as its phenotypical behavior, we are perfectly equipped to classify all the observations in this new research area.

The primary focus of these preliminary experiments on the exosomal secretion was to determine the number of secreted vesicles, their size distribution, and their population regarding the tetraspanin surface expression. These parameters would be a first indication of changes in cellular cross-talk under microgravity, and hence, would point to an adaptation of the cells to the change in environment. The broad majority of captured particles are exosomes per size definition, in the range of 50–100 nm [48,49,54]. Comparing the two sample sets, we can see a definite increase in the overall particle number in the FMs in reaction to the microgravity exposure. The relative size distribution stays comparatively the same, and the mode of particle sizes is still 60 nm, even though the total number on all the levels rose in the flight samples. The logical assumption is that, at a minimum, the amount of transferred cargo from the tumor cell to the target cell increases proportionally to the larger number of secreted vesicles, even though the actual content of exosomes can depend on various other intra- and extracellular conditions [67,71,72]. In consequence, our working hypothesis was that the change in gravitational forces should have a distinct effect on the exosome number, as well as their surface protein and cargo composition mirroring the adaptions of the cells, we have observed in this and previous experiments. This study in fact showed that the number of vesicles and the composition of sub-populations regarding their surface tetraspanin composition does considerably change under the influence of real microgravity.

The analysis by fluorescence of the EVs counterstained with the tetraspanins CD9, CD63, and CD81 further supports the previous interferometric results and allows for the detection of vesicles that fall below the lower detection limit of 50 nm allowed by the interferometric measurement. Here, though, the changes in the surface proteins expressed on the captured exosomes also exhibit a change in the sub-populations present in the GM and FM sample sets. Tetraspanins are not only valuable biomarkers that are enriched in the membranes of exosomes, they are a superfamily of proteins involved in a multitude of biological processes. Those processes include cell adhesion, motility, invasion, and membrane fusion, as well as signaling and protein trafficking [73,74]. Tetraspanins form a network with exosomal and other proteins regulating signaling transduction pathways and the formation of premetastatic sites in the tumor microenvironment (TME) [75]. Tetraspanins also play a critical role in the target cell selection for exosome uptake and, therefore, the reprogramming of the target cell. CD9, CD63, and CD81, in conjunction with other tetraspanins, promote (in association with exosomes) cancer cell motility, invasion, metastasis, tumor initiation, promotion, progression, and angiogenesis [74,75].

In the TME, CD9 suppresses motility and promotes adherence to the surrounding matrix which subsequently leads to a suppression of tumor progression. The CD9 downregulation has been correlated with tumor progression, and is often found in late-stage tumors [76]. On the other hand, CD9 has been observed to display converse activity, and therefore, it is difficult to pinpoint a specific role to this tetraspanin in regards to an increase or decrease of tumorigenicity as a result of exposure to real microgravity [76]. Additionally, CD9 is emerging as an important regulator controlling the expression and activity of different adhesion molecules, among them several metalloproteinases [77,78,79,80]. Riwaldt et al. described the gene alterations of the matrix metalloproteinases MMP3 and MMP9 and the resulting phenotypical changes in another human thyroid cancer cell line (UCLA RO 82-W-1). In this article, the authors discuss a study of pathways involved in the transition from a two-dimensional to a three-dimensional cell growth as a result of the simulated microgravity [25].

CD63 and CD81 have been shown to be important contributors in the sorting of protein cargo to EVs. Wnt11 aids cell migration and metastasis in breast cancer cells, and the sorting of the Wnt11 cargo to EVs is supported by CD81, but not other tetraspanins [40,73]. Similarly, CD63 is a key contributor to the protein loading of intraluminal vesicles (ILVs) in the MVBs during the exosome biogenesis [73,81]. In cancer, the surface expression of CD63 is linked to tumor cell motility, and a decrease of this tetraspanin is linked to the increased malignancy and higher chances of metastasis [82,83]. The results of the CellBox-1 experiments show a high expression of CD63, with an additional increase with exposure to microgravity. Whether this leads to changes in the tumorigenic behavior of FTC-133 or in the cargo of the secreted exosomes will have to be determined in further studies. Further, CD81 has been found to contribute to tumor progression and an enhanced metastatic phenotype [84,85,86,87]. As with CD63, at this stage of the investigation, one cannot attribute a particular change in function to the observed changes without further experimentation.

Co-expression of CD9/CD81 was shown to regulate TGF-β1 signaling in melanoma by providing critical support for the TGFβR2-TGFβR1 association, which in turns favors epithelial to mesenchymal transition-like changes, invasion, and metastases [87]. In this study, we have shown an increase in the CD9/CD81 population in the FMs compared to GMs, but whether this could point to a transition of a more aggressive stage in the FTC-133 cells is another hypothesis that requires further data in order to be adequately discussed.

To conclude, it would certainly be very interesting to evaluate the changes in the exosomal proteomic cargo following prolonged exposure to microgravity in comparison to the results gained from the proteomic analysis of the FTC-133 cells of the CellBox-1 mission [23,24]. This would give an even deeper insight into the cellular regulation post-transcription and could not only enhance our knowledge of these pathways, but also point towards a direction for future treatment regimens. This demonstrates the vast possibilities promised by EVs in the medical field, and is surely worth further exploration.

## 4. Materials and Methods

### 4.1. Cell Cultures

Human follicular thyroid cancer cells (FTC-133), purchased from the Health Protection Agency Culture Collections (HPACC, Salisbury, UK), were cultured in RPMI-1640 (Invitrogen, Eggenstein, Germany), supplemented with 10% fetal calf serum (FCS, Biochrom, Berlin, Germany), penicillin (100 U/mL; Merck Millipore), and streptomycin (100 µg/mL; Merck Millipore) at 37 °C and 5% CO_2_, as described previously. Prior to the start of the experiment, 10^6^ cells were loaded into an automated cell culture system, the FM hardware [22], or alternatively seeded in T-25 culture flasks for the ground control experiments. The medium exchange and cell fixation at the experimental endpoint occurred automatically as scheduled [23].

### 4.2. CellBox-1 Spaceflight Experiment

**Figure 8:**
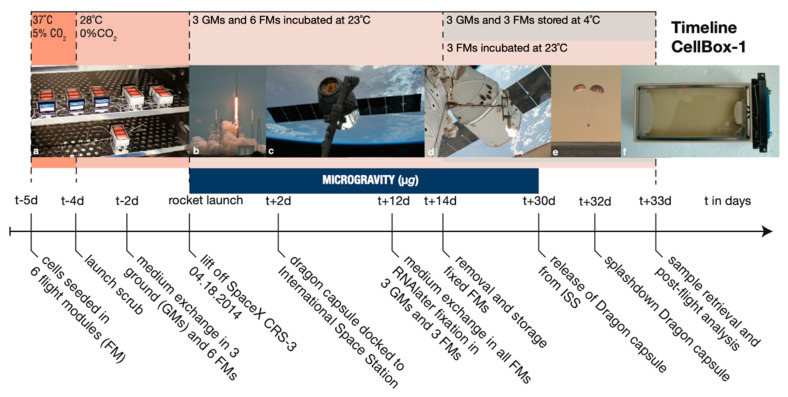
Timeline of the CellBox-1 experiment. (**a**) Six flight modules (FMs) incubated at 37 °C, 5% CO_2_. Each container filled with 1 × 10^6^ FTC-133 thyroid cancer cells. (**b**) Falcon-9 lift-off within Cargo Resupply Services mission 3 (SpaceX CRS-3). (**c**) SpaceX Dragon capsule arrives at the International Space Station (ISS). (**d**) Dragon capsule docked to ISS. (**e**) Dragon capsule splashdown into the Pacific Ocean. (**f**) Flight module prior to sample collection and post-flight analysis. (Picture a courtesy of RaisFoto, pictures **b**–**e** photo credit SpaceX).

The CellBox-1 spaceflight experiment was part of the SpaceX CRS-3 Commercial Resupply Service mission, launched on 18 April, 2014 (https://www.dlr.de/content/de/bilder/2014/2/start-des-des-cellbox-experiments_15157.html (accessed on 20 February 2021)) and is reported in detail by Riwaldt et al. [23]. In brief, 1 × 10^6^ FTC-133 was seeded in nine cell containers (FM) and incubated at 37 °C and 5% CO_2_. 24 h prior to launch, three FMs were transferred to an incubation chamber at 23 °C at Cape Canaveral to serve as ground controls (GM). The remaining six FMs were moved to the Dragon capsule and flown to the International Space Station (ISS). Upon arrival at the ISS, the FMs were incubated continuously at 23 °C at a *g*-force oscillating around ± 0.005 g. After 12 days of microgravity, the cells of three FMs were fixed using RNA*later* via an automated process [22], collecting the aspirated media. The remaining three FMs were continuously incubated at 23 °C for the remainder of the spaceflight and therefore, not included in this analysis. The GMs were treated identically and simultaneously on the ground. 48 h post-fixation, both the FMs and GMs were cooled to 4 °C. After the return of the Dragon vessel on 20 May 2014, all the containers were returned to the lab for cell and cell supernatant harvest (Figure 8).

### 4.3. Exosome Harvest and Isolation

After the harvest, the cell supernatants of the FMs and GMs were subjected to an adjusted differential centrifugation protocol [88] using a swinging bucket rotor, consisting of two consecutive spins at 300× *g* (10 min, 4 °C) followed by centrifugation at 2500× *g* (15 min, 4 °C, twice) to pellet cells, cell debris, and large vesicles. High speed centrifugation was not necessary as the analysis via ExoView® does not require a preceding particle isolation. The collected supernatants were divided into 2 mL aliquots and stored at −80 °C until further analysis.

### 4.4. ExoView^®^ Kit Assay Procedure

For the capture and analysis of the CellBox-1 supernatants, the EV-TETRA-C ExoView Tetraspanin Kit (NanoView Biosciences, Boston, MA, USA) was used, with all samples processed and stained according to the manufacturer’s protocol. The spots on the chip plates were coated with antibodies for the tetraspanins CD9, CD81, and CD63, as well as an IgG negative control, all in triplicates. In short, the samples were diluted 1:2 and incubated ON at RT on the sealed chip plate in order to capture the exosomes present. The incubation was followed by three wash steps prior to the surface membrane immunofluorescence staining. An antibody mixture containing fluorescently labeled anti-CD9 (CF^®^ 488A), anti-CD81 (CF^®^ 555), and anti-CD63 (CF^®^ 647) was pipetted onto the chip and incubated for 1 h on an orbital shaker, blocked from light exposure. Three wash steps with the supplied wash buffer and two washes with DI water for 3 min each (RT, shaking, light omitted) followed this incubation. Subsequently, the chip plates were dried and scanned as described below.

### 4.5. Digital Detection of Exosomes

The chip plates were scanned using the ExoView R100 (NanoView Biosciences, Boston, MA, USA) in combination with the ExoScan software. All the chip plates used were pre-scanned prior to the exosome capture to obtain a baseline signal, then the exosome-laden chips were scanned identically positioned on the stage platform. The analysis was visualized with the ExoView software, resulting in the EV count, size distribution, and colocalization of EV subpopulations.

### 4.6. Statistical Analysis

The total counts of numbers were analyzed via the unpaired *t*-test, comparing all the samples from either the GM or FM group respective to the capture spot on the chip plate, CD9, CD63, or CD81. The analysis was conducted using the GraphPad Prism 9 software (GraphPad Software, San Diego, CA, USA).

## 5. Conclusions

This initial analysis of secreted EVs harvested from human follicular thyroid cells that were either subjected to real microgravity on the ISS or grown on Earth is a first step towards evaluating the feasibility of future experiments with exosomes, and the range of questions that such experiments could answer. The changes and adaptations we have observed promise to enhance our understanding of the changes and adaptations of the human organism to microgravity exposure on a cellular level. Additionally, these studies may offer new biomarkers and therapeutic approaches for multiple clinical pictures.

## Figures and Tables

**Table 1:** Particle size distribution baseline corrected, GM sample set.

		CD81			CD63			CD9	
Size (nm)	GM-1	GM-2	GM-3	GM-1	GM-2	GM-3	GM-1	GM-2	GM-3
50	4.3	41.7	−16.7	204	179	172.3	696.7	690.3	633.6
55	−18	48.4	−32.3	187	175	193	1536	1136.7	1335.7
60	5.4	26.3	32.4	120.7	103	92	1162.7	628.6	983.7
65	31	38.7	37.7	45	83.7	44.7	784.7	376.3	663.4
70	33.4	15	27.6	30.2	54.3	17.6	520	259.7	426
75	22.7	23.7	23	19.2	44	16	348	194	327
80	17	20.3	15.7	11.3	38.7	9.7	271.6	156.3	248.4
85	13.7	22.3	13.3	9	30.3	7	199	121	184.3
90	13	27.7	9.3	5.3	28.3	8.7	142.6	85	138
95	7.3	10	9	4.5	19.4	5	102	65	92.3
100	8.7	10.3	6	5.5	18.3	7.4	64.3	52.3	64.4
105	6.7	6.7	3.3	6.5	4.3	3.6	42.3	30	56.3
110	5.6	9	3	3.8	6.6	2.3	41	18.3	35
115	6	14.6	3.4	3.3	7.3	1.4	22	14.6	21
120	5	7.3	3.3	0.8	6.3	3.3	18.3	8.7	17.7
125	2	6.7	2.4	1.8	7.4	1.4	10.3	7	12.4
130	3.4	6.7	2.3	0.2	6.4	1.7	7.7	5.4	9
135	1.7	3	1.3	1	1	−0.3	6	6	8.3
140	3	7	0.4	0.5	5	1	5.7	3	3.7
145	1.3	2.6	0.7	0	−1.7	1	4.7	0.6	4.4
150	1	1.3	0.3	0	2.7	0.7	3.7	2	3
155	1	3.6	1	−0.7	0.3	0.3	1	0.3	4.3
160	0.3	2.3	0.3	0	2.3	0.3	1.3	1	1.7
165	0.3	3	0.3	1.5	3.3	0	1.7	2.3	1
170	0.3	3.4	1.7	0.8	1	0.3	1.6	2.4	1.7
175	1	1.3	0.7	0	1	0	0.3	1.3	0.7
180	0	2	0.7	−0.3	1.3	0	0.7	2	0
185	0.3	2.7	0	0	1.7	0	0	1.4	1
190	0	3	0.3	0	1.6	0	0	0.6	0.7
195	0.4	−0.3	0	−0.3	1.4	0	−0.3	−0.3	0.3
200	−0.3	1.3	1	−0.3	1	0.7	0.4	0.3	0.7

**Table 2:** Particle size distribution baseline corrected, FM sample set.

		CD81			CD63			CD9	
Size (nm)	GM-1	GM-2	GM-3	GM-1	GM-2	GM-3	GM-1	GM-2	GM-3
50	4.3	41.7	−16.7	204	179	172.3	696.7	690.3	633.6
55	−18	48.4	−32.3	187	175	193	1536	1136.7	1335.7
60	5.4	26.3	32.4	120.7	103	92	1162.7	628.6	983.7
65	31	38.7	37.7	45	83.7	44.7	784.7	376.3	663.4
70	33.4	15	27.6	30.2	54.3	17.6	520	259.7	426
75	22.7	23.7	23	19.2	44	16	348	194	327
80	17	20.3	15.7	11.3	38.7	9.7	271.6	156.3	248.4
85	13.7	22.3	13.3	9	30.3	7	199	121	184.3
90	13	27.7	9.3	5.3	28.3	8.7	142.6	85	138
95	7.3	10	9	4.5	19.4	5	102	65	92.3
100	8.7	10.3	6	5.5	18.3	7.4	64.3	52.3	64.4
105	6.7	6.7	3.3	6.5	4.3	3.6	42.3	30	56.3
110	5.6	9	3	3.8	6.6	2.3	41	18.3	35
115	6	14.6	3.4	3.3	7.3	1.4	22	14.6	21
120	5	7.3	3.3	0.8	6.3	3.3	18.3	8.7	17.7
125	2	6.7	2.4	1.8	7.4	1.4	10.3	7	12.4
130	3.4	6.7	2.3	0.2	6.4	1.7	7.7	5.4	9
135	1.7	3	1.3	1	1	−0.3	6	6	8.3
140	3	7	0.4	0.5	5	1	5.7	3	3.7
145	1.3	2.6	0.7	0	−1.7	1	4.7	0.6	4.4
150	1	1.3	0.3	0	2.7	0.7	3.7	2	3
155	1	3.6	1	−0.7	0.3	0.3	1	0.3	4.3
160	0.3	2.3	0.3	0	2.3	0.3	1.3	1	1.7
165	0.3	3	0.3	1.5	3.3	0	1.7	2.3	1
170	0.3	3.4	1.7	0.8	1	0.3	1.6	2.4	1.7
175	1	1.3	0.7	0	1	0	0.3	1.3	0.7
180	0	2	0.7	−0.3	1.3	0	0.7	2	0
185	0.3	2.7	0	0	1.7	0	0	1.4	1
190	0	3	0.3	0	1.6	0	0	0.6	0.7
195	0.4	−0.3	0	−0.3	1.4	0	−0.3	−0.3	0.3
200	−0.3	1.3	1	−0.3	1	0.7	0.4	0.3	0.7

## Data Availability

Not applicable.

## Supplementary Materials

The following are available online at https://www.mdpi.com/1422-0067/22/4/2132/s1. Figure S1: Total count of captured particles via the interferometric analysis; Table S1: Absolute values of colocalization analysis; Table S2: Colocalization analysis calculated as percentages.

## Abbreviations

AB	Antibody
EMT	Epithelial to Mesenchymal Transition
EV	Extracellular Vesicle
GM	Ground Module
FM	Flight Module
ILV	Intraluminal Vesicle
MVBs	Multivesicular Bodies
NTA	Nanoparticle Tracking Analysis
ON	Overnight
RPM	Random Positioning Machine
RT	Room temperature
SD	Standard Deviation
SP-IRIS	Single Particle Interferometric Reflectance Imaging Sensor
TME	Tumor Microenvironment
