# Supplementary material for: Changes in Exosome Release in Thyroid Cancer Cells after Prolonged Exposure to Real Microgravity in Space
Source: Int J Mol Sci. 2021 Feb 21;22(4):2132. doi: 10.3390/ijms22042132 (PMC7924847; doi:10.3390/ijms22042132)
Supplement: Supplementary file 1 [file ijms-22-02132-s001.pdf]

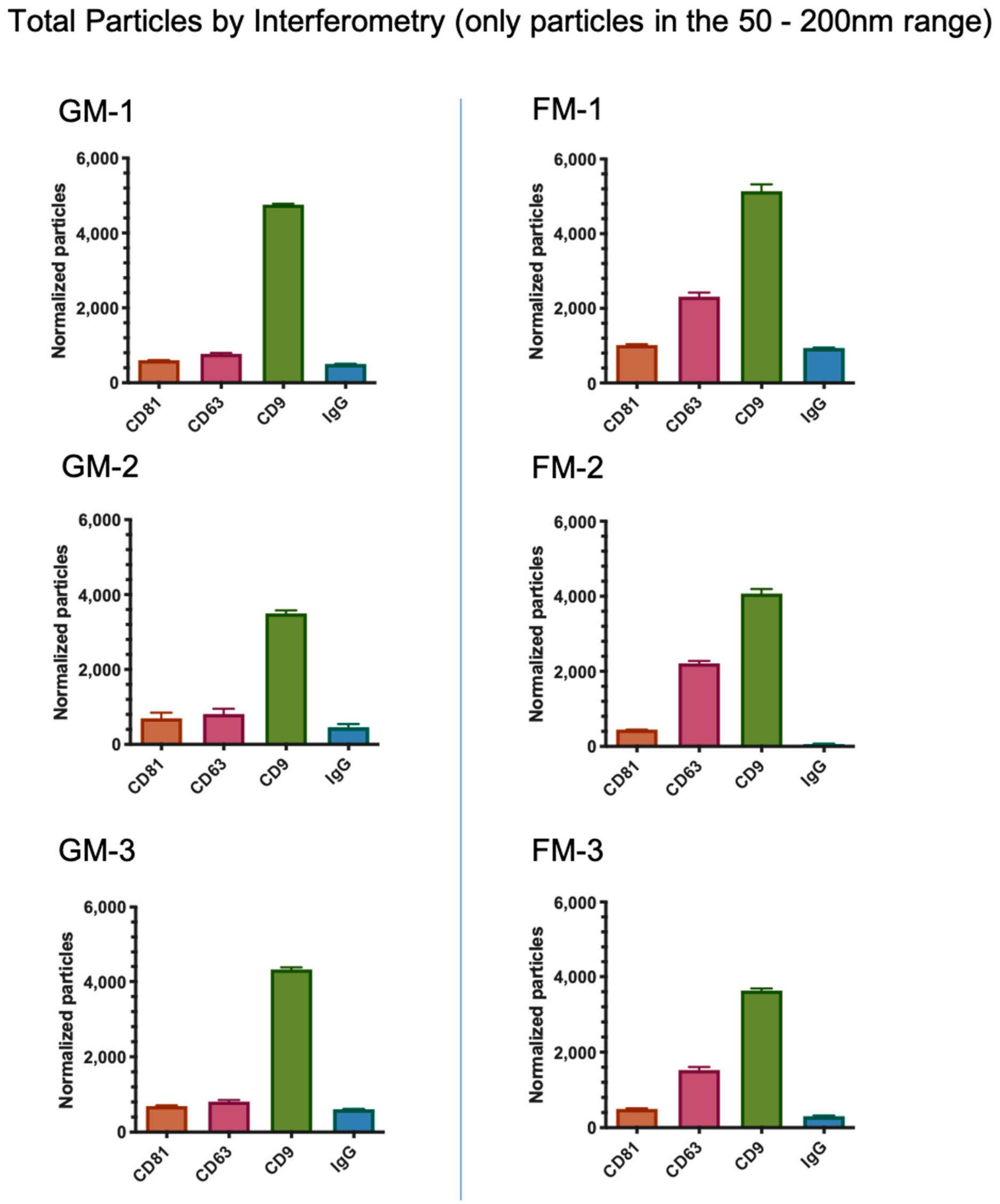

Figure S1. Total count of captured particles via interferometric analysis, from both sample sets, GFM and FFM, values are shown with SD and include the IgG control spot.

Table S1. Absolute values of colocalization analysis: both sample sets were measured in triplicates, displayed in mean values and SD.

GFM-1	CD81		CD9		CD63		CD9/63		CD9/81		CD63/81		CD9/81/63	
	**Mean**	**SD**	**Mean**	**SD**	**Mean**	**SD**	**Mean**	**SD**	**Mean**	**SD**	**Mean**	**SD**	**Mean**	**SD**
**CD81 spot**	463.5	21.2	0.0	0.0	0.0	0.0	0.0	0.0	2445.2	123.1	733.7	14.1	363.7	7.7
**CD63 spot**	0.0	0.0	0.0	0.0	3644.8	50.5	497.9	46.1	0.0	0.0	501.0	3.5	146.2	18.7
**CD9 spot**	0.0	0.0	4009.7	399.8	0.0	0.0	1025.9	33.5	2766.0	156.9	0.0	0.0	410.9	51.6

GFM-2	CD81		CD9		CD63		CD9/63		CD9/81		CD63/81		CD9/81/63	
	**Mean**	**SD**	**Mean**	**SD**	**Mean**	**SD**	**Mean**	**SD**	**Mean**	**SD**	**Mean**	**SD**	**Mean**	**SD**
**CD81 spot**	405.4	13.6	0.0	0.0	0.0	0.0	0.0	0.0	1960.0	68.3	455.4	21.8	155.3	8.0
**CD63 spot**	0.0	0.0	0.0	0.0	3303.3	209.2	380.9	33.8	0.0	0.0	348.0	36.2	96.9	15.6
**CD9 spot**	0.0	0.0	2168.5	68.1	0.0	0.0	648.8	17.7	2724.8	94.2	0.0	0.0	216.4	7.2

GFM-3	CD81		CD9		CD63		CD9/63		CD9/81		CD63/81		CD9/81/63	
	**Mean**	**SD**	**Mean**	**SD**	**Mean**	**SD**	**Mean**	**SD**	**Mean**	**SD**	**Mean**	**SD**	**Mean**	**SD**
**CD81 spot**	474.8	17.9	0.0	0.0	0.0	0.0	0.0	0.0	2014.5	44.2	537.1	21.9	245.1	25.0
**CD63 spot**	0.0	0.0	0.0	0.0	3525.9	121.4	441.6	30.8	0.0	0.0	444.4	44.0	146.4	9.3
**CD9 spot**	0.0	0.0	3329.7	67.8	0.0	0.0	733.2	18.4	2550.2	44.2	0.0	0.0	329.5	12.0

FFM-1	CD81		CD9		CD63		CD9/63		CD9/81		CD63/81		CD9/81/63	
	**Mean**	**SD**	**Mean**	**SD**	**Mean**	**SD**	**Mean**	**SD**	**Mean**	**SD**	**Mean**	**SD**	**Mean**	**SD**
**CD81 spot**	1108.7	19.5	0.0	0.0	0.0	0.0	0.0	0.0	2310.4	70.6	796.9	41.5	401.0	26.0
**CD63 spot**	0.0	0.0	0.0	0.0	7960.4	425.3	487.8	59.5	0.0	0.0	674.5	59.9	242.5	36.7
**CD9 spot**	0.0	0.0	2857.6	216.8	0.0	0.0	932.7	42.4	3838.6	147.7	0.0	0.0	556.8	30.5

FFM-2	CD81		CD9		CD63		CD9/63		CD9/81		CD63/81		CD9/81/63	
	**Mean**	**SD**	**Mean**	**SD**	**Mean**	**SD**	**Mean**	**SD**	**Mean**	**SD**	**Mean**	**SD**	**Mean**	**SD**
**CD81 spot**	1270.1	62.3	0.0	0.0	0.0	0.0	0.0	0.0	3209.0	298.5	920.1	15.5	497.1	69.5
**CD63 spot**	0.0	0.0	0.0	0.0	7745.2	118.2	625.0	55.5	0.0	0.0	637.4	34.0	224.5	20.0
**CD9 spot**	0.0	0.0	2853.4	79.5	0.0	0.0	1148.7	44.3	4344.0	61.2	0.0	0.0	632.0	45.6

FFM-3	CD81		CD9		CD63		CD9/63		CD9/81		CD63/81		CD9/81/63	
	**Mean**	**SD**	**Mean**	**SD**	**Mean**	**SD**	**Mean**	**SD**	**Mean**	**SD**	**Mean**	**SD**	**Mean**	**SD**
**CD81 spot**	1215.3	101.3	0.0	0.0	0.0	0.0	0.0	0.0	2523.6	295.0	802.2	30.9	349.0	53.6
**CD63 spot**	0.0	0.0	0.0	0.0	7862.5	1.4	404.5	39.5	0.0	0.0	595.4	72.3	169.4	21.5
**CD9 spot**	0.0	0.0	2376.9	277.9	0.0	0.0	912.5	67.3	4252.4	166.9	0.0	0.0	532.8	47.1

Table S2. Colocalization analysis calculated as percentages: both sample sets were measured in triplicates, displayed in mean values and SD.

GFM-1	CD81		CD9		CD63		CD9/63		CD9/81		CD63/81		CD9/81/63	
	Mean	SD	Mean	SD	Mean	SD	Mean	SD	Mean	SD	Mean	SD	Mean	SD
CD81 spot	11.6	0.4	0.0	0.0	0.0	0.0	0.0	0.0	61.0	1.1	18.3	0.6	9.1	0.5
CD63 spot	0.0	0.0	0.0	0.0	76.1	0.8	10.4	0.7	0.0	0.0	10.5	0.2	3.0	0.3
CD9 spot	0.0	0.0	48.8	3.6	0.0	0.0	12.5	0.5	33.7	2.7	0.0	0.0	5.0	0.7

GFM-2	CD81		CD9		CD63		CD9/63		CD9/81		CD63/81		CD9/81/63	
	Mean	SD	Mean	SD	Mean	SD	Mean	SD	Mean	SD	Mean	SD	Mean	SD
CD81 spot	13.6	0.4	0.0	0.0	0.0	0.0	0.0	0.0	65.9	1.2	15.3	0.8	5.2	0.1
CD63 spot	0.0	0.0	0.0	0.0	80.0	0.6	9.2	0.4	0.0	0.0	8.4	0.3	2.3	0.2
CD9 spot	0.0	0.0	37.7	0.7	0.0	0.0	11.3	0.0	47.3	0.6	0.0	0.0	3.8	0.1

GFM-3	CD81		CD9		CD63		CD9/63		CD9/81		CD63/81		CD9/81/63	
	Mean	SD	Mean	SD	Mean	SD	Mean	SD	Mean	SD	Mean	SD	Mean	SD
CD81 spot	14.5	0.5	0.0	0.0	0.0	0.0	0.0	0.0	61.6	1.4	16.4	0.7	7.5	0.7
CD63 spot	0.0	0.0	0.0	0.0	77.4	0.5	9.7	0.5	0.0	0.0	9.7	0.7	3.2	0.1
CD9 spot	0.0	0.0	48.0	0.4	0.0	0.0	10.6	0.1	36.7	0.1	0.0	0.0	4.7	0.2

FFM-1	CD81		CD9		CD63		CD9/63		CD9/81		CD63/81		CD9/81/63	
	Mean	SD	Mean	SD	Mean	SD	Mean	SD	Mean	SD	Mean	SD	Mean	SD
CD81 spot	24.0	0.8	0.0	0.0	0.0	0.0	0.0	0.0	50.0	1.2	17.3	0.8	8.7	0.4
CD63 spot	0.0	0.0	0.0	0.0	85.0	2.0	5.2	0.8	0.0	0.0	7.2	0.8	2.6	0.5
CD9 spot	0.0	0.0	34.9	2.0	0.0	0.0	11.4	0.2	46.9	1.9	0.0	0.0	6.8	0.2

FFM-2	CD81		CD9		CD63		CD9/63		CD9/81		CD63/81		CD9/81/63	
	Mean	SD	Mean	SD	Mean	SD	Mean	SD	Mean	SD	Mean	SD	Mean	SD
CD81 spot	21.6	1.9	0.0	0.0	0.0	0.0	0.0	0.0	54.4	2.1	15.6	0.7	8.4	0.7
CD63 spot	0.0	0.0	0.0	0.0	83.9	1.1	6.8	0.6	0.0	0.0	6.9	0.4	2.4	0.2
CD9 spot	0.0	0.0	31.8	0.4	0.0	0.0	12.8	0.2	48.4	0.5	0.0	0.0	7.0	0.4

FFM-3	CD81		CD9		CD63		CD9/63		CD9/81		CD63/81		CD9/81/63	
	Mean	SD	Mean	SD	Mean	SD	Mean	SD	Mean	SD	Mean	SD	Mean	SD
CD81 spot	24.9	2.0	0.0	0.0	0.0	0.0	0.0	0.0	51.5	2.2	16.4	0.8	7.1	0.5
CD63 spot	0.0	0.0	0.0	0.0	87.1	1.3	4.5	0.4	0.0	0.0	6.6	0.7	1.9	0.2
CD9 spot	0.0	0.0	29.4	2.5	0.0	0.0	11.3	0.5	52.7	1.9	0.0	0.0	6.6	0.3
